# Implementing a video call visit system in a coronavirus disease 2019 unit

**DOI:** 10.4102/phcfm.v12i1.2637

**Published:** 2020-09-15

**Authors:** Muhammad S. Moolla, Alistair Broadhurst, Mohammed A. Parker, Arifa Parker, Abdurasiet Mowlana

**Affiliations:** 1 Division of General Medicine, Department of Medicine, Faculty of Medicine and Health Sciences, Tygerberg Hospital, Stellenbosch University, Cape Town, South Africa; 2 Divisions of Infectious Diseases and General Medicine, Department of Medicine, Faculty of Medicine and Health Sciences, Tygerberg Hospital, Stellenbosch University, Cape Town, South Africa

**Keywords:** communication, digital, mobile technology, video call, COVID-19, coronavirus, patient experience, social distancing

## Abstract

The lockdown and physical distancing strategies imposed to combat COVID-19 have caused seismic shifts at all levels of society. Hospitals have been particularly affected. Healthcare workers (HCW’s) wore PPE during all patient interactions and visitors were prohibited. Life for a patient became lonelier and for those with severe acute respiratory syndrome coronavirus 2 (SARS-CoV-2) measures were even more severe. HCW’s must treat patients following a biopsychosocial approach and promote communication between patients and loved ones. We implemented a low cost Video Call Visit system at Tygerberg Hospital, Cape Town. In this article we discuss the elements of a successful implementation and potential pitfalls in the context of a pandemic, notably cross-infection and privacy. Rapid but responsible innovation using 21st century tools was required to address the many challenges of the pandemic, including improving the lived experience for patients and families. These should be intended to last after the pandemic has passed.

## Introduction

Following the emergence of the severe acute respiratory syndrome coronavirus 2 (SARS-CoV-2) pandemic, a national state of disaster was declared in South Africa on 15 March 2020. Social distancing measures were implemented to ‘flatten the curve’ to a level manageable by available healthcare resources.^1^ In hospitals, this has led to many changes, including the prohibition of visitation and the use of personal protective equipment (PPE), such as masks and face shields by the staff.^2^ These measures prevent the spread of infection amongst the staff and patients, but for the individual hospitalisation is lonelier than ever. There is no visiting hour to look forward to, and the interaction with the staff is buried behind the layers of plastic, the face is barely visible.

**FIGURE 1:**
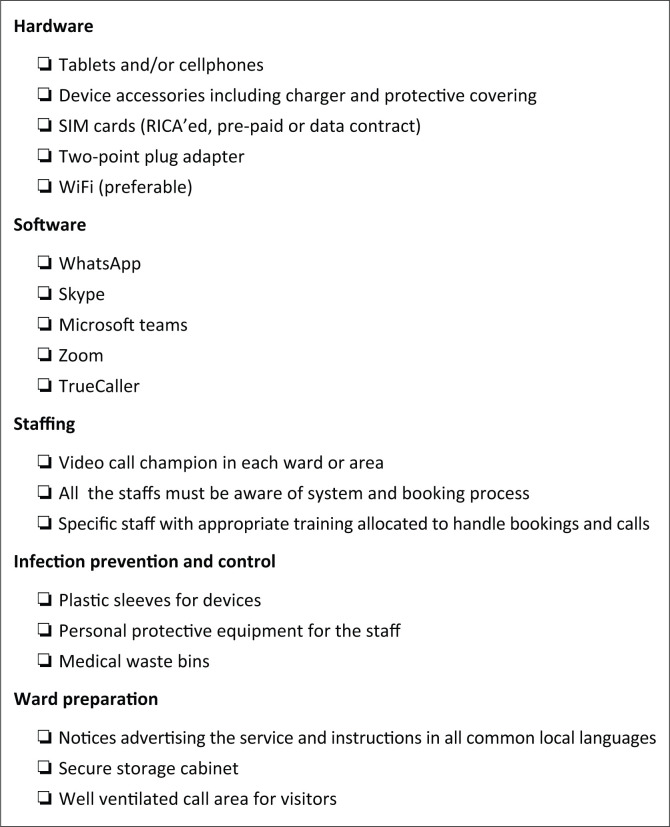
Checklist of requirements for implementing a video call visit system.

In the absence of a cure for coronavirus disease 2019 (COVID-19), we must continue to look after patients following a biopsychosocial approach. Modern techniques promoting communication between patients and loved ones should be encouraged. At Tygerberg Hospital (TBH), we implemented a video call visit (VCV) system in the COVID-19 Unit to safely reconnect patients with loved ones. There are several components required as shown in the checklist (Figure 1). We discuss these in the context of the current pandemic.

## Discussion

We received donations of tablets and purchased prepaid SIM cards. The hospital, attached to Stellenbosch University, has high-speed internet available via WiFi in all COVID-19 wards following their preparatory refurbishment. At institutions without ready internet access, SIM cards with data contracts are a more costly alternative.^3^ We prepared the devices with several popular applications for video calls (Figure 1).

At TBH, the staff takes responsibility for individual COVID-19 wards. This minimises contact between the staff and shared surfaces.^2^ A separate device was allocated to each ward. To promote awareness of the VCV, we placed posters in English, Afrikaans and Xhosa at the entrances and appointed a Video Call Champion (VCC) in each ward. The VCC’s role includes raising awareness of the system and taking primary responsibility for making calls. Critically ill patients requiring transfer to intensive care unit (ICU) and those identified for palliative care are prioritised and strongly encouraged to make use of the VCV.

The appointment of champions is a crucial step in ensuring the success of a system such as VCV. When selecting these individuals, ideal characteristics include (1) having positional or personal authority in the ward which (2) they can wield in a non-traditional manner, (3) a willingness to use that influence to benefit the system and (4) the desire to go above and beyond their expected responsibilities. They must ensure broad-based acceptance of VCV, maintaining a clear sense of purpose amongst the rest of the team and provide motivation to implement the system.^4^ In our unit, this role was filled by junior doctors.

Three pathways exist for initiating calls. The first is patient-initiated, either *de novo* or after an offer by the staff. Family members may book calls by calling the nursing station or sending a message to the VCV device and providing patient name and ward, contact name and number and username for the preferred application. Calls were restricted to specific hours to limit the impact on clinical duties with exceptions made for special cases.

Two major concerns exist with the use of a shared electronic device. One is contamination of devices and cross-infection to the staff and other patients. SARS-CoV-2 can remain viable for up to 3 days on the plastic surfaces of electronic devices.^5^ Measures taken to prevent this are the use of disposable plastic sleeves to cover the device with each use,^6^ decontamination with an alcohol-based solution between patients^7^ and HCW’s wearing standard PPE.

Privacy is also a concern. Messages and shared media are stored on the device and must be deleted after each use. All accounts must be logged out. The staff should receive in-service training to ensure that patients are properly protected.^8^

Implementation of VCV is important in promoting equity for patients. Some patients can undoubtedly do this for themselves. Others do not have the financial, technical or physical means to communicate independently via social media and must not be forgotten. Family members and friends may equally not have access to these facilities. At TBH, we used additional devices to allow loved ones to participate from a safe location on the hospital premises.

South Africa is a country where transport cost and time often prohibit regular visits from loved ones. Implementing VCV can have legacy benefits providing relief to patients and families after the pandemic has passed.

## Conclusion

Rapid but responsible innovation is required to navigate the many new challenges posed by the SARS-CoV-2 pandemic. The ubiquity, ever expanding utility and connectedness of electronic devices make them ideal in this respect. Previously seen as distracting and anti-social, in this time of pandemic they are one of the few tools we have to reconnect and express our humanity.

Implementation of VCV can be done inexpensively and regardless of the existing level of supporting infrastructure. The staff must remain alert to potential pitfalls, particularly cross-contamination and breaches of privacy. The goal must be to establish these tools as the standard of care going forward.

As HCW’s, it is our responsibility to help reconnect COVID-19 patients with their loved ones. VCV is only one possibility. The current policy disallowing hospital visits should be challenged. Close family members could, while wearing PPE and after discussion and understanding of the risks, visit under strict conditions. Further research is required to the safety of such a concession.
